# Development Technologies for the Monitoring of Six-Minute Walk Test: A Systematic Review

**DOI:** 10.3390/s22020581

**Published:** 2022-01-12

**Authors:** Ivan Miguel Pires, Hanna Vitaliyivna Denysyuk, María Vanessa Villasana, Juliana Sá, Diogo Luís Marques, José Francisco Morgado, Carlos Albuquerque, Eftim Zdravevski

**Affiliations:** 1 Instituto de Telecomunicações, Universidade da Beira Interior, 6200-001 Covilhã, Portugal; hanna.denysyuk@av.it.pt; 2 Escola de Ciências e Tecnologia, University of Trás-os-Montes e Alto Douro, Quinta de Prados, 5001-801 Vila Real, Portugal; 3 Centro Hospitalar Universitário da Cova da Beira, 6200-251 Covilhã, Portugal; 72152@chbv.min-saude.pt; 4 Health Sciences Research Unit: Nursing (UICISA: E), School of Health, Polytechnic Institute of Viseu, 3504-510 Viseu, Portugal; julianasa@fcsaude.ubi.pt (J.S.); calbuquerque@essv.ipv.pt (C.A.); 5 Faculty of Health Sciences, Universidade da Beira Interior, 6200-506 Covilhã, Portugal; 6 Centro Hospitalar Universitário do Porto, 4099-001 Oporto, Portugal; 7 Department of Sport Sciences, University of Beira Interior, 6201-001 Covilhã, Portugal; diogo.marques@ubi.pt; 8 Computer Science Department, Polytechnic Institute of Viseu, 3504-510 Viseu, Portugal; fmorgado@estgv.ipv.pt; 9 Faculty of Computer Science and Engineering, University Ss Cyril and Methodius, 1000 Skopje, North Macedonia; eftim.zdravevski@finki.ukim.mk

**Keywords:** six-minute walk test, IoT, systematic review, mobile devices, telemedicine

## Abstract

In the pandemic time, the monitoring of the progression of some diseases is affected and rehabilitation is more complicated. Remote monitoring may help solve this problem using mobile devices that embed low-cost sensors, which can help measure different physical parameters. Many tests can be applied remotely, one of which is the six-minute walk test (6MWT). The 6MWT is a sub-maximal exercise test that assesses aerobic capacity and endurance, allowing early detection of emerging medical conditions with changes. This paper presents a systematic review of the use of sensors to measure the different physical parameters during the performance of 6MWT, focusing on various diseases, sensors, and implemented methodologies. It was performed with the PRISMA methodology, where the search was conducted in different databases, including IEEE Xplore, ACM Digital Library, ScienceDirect, and PubMed Central. After filtering the papers related to 6MWT and sensors, we selected 31 papers that were analyzed in more detail. Our analysis discovered that the measurements of 6MWT are primarily performed with inertial and magnetic sensors. Likewise, most research studies related to this test focus on multiple sclerosis and pulmonary diseases.

## 1. Introduction

The six-minute walk test (6MWT) was officially introduced in 2002 by the American Thoracic Society, providing a detailed guideline for performing, monitoring, and analyzing the physical conditions of the patients [1]. It is a sub-maximal exercise test used to assess aerobic capacity and endurance. Changes in performance capacity are determined based on the distance covered in six minutes [1,2]. The 6MWT is helpful to evaluate performance and identify clinical conditions in preschool children (2–5 years), children (6–12 years), adults (18–64 years), and older adults (65+) with a wide range of diagnoses [3,4,5]. Even though the test was initially used to assess patients with cardiopulmonary issues, it was introduced over time in numerous other conditions [6,7]. It evaluates the individual’s functional capacity and provides valuable information regarding all the systems during physical activity, including pulmonary and cardiovascular systems, blood circulation, neuromuscular units, body metabolism, and peripheral circulation [8,9]. The 6MWT is used for different health conditions, including arthritis, fibromyalgia, geriatrics, multiple sclerosis, Parkinson’s disease, spinal cord injury, stroke, muscle disorders, spinal muscular atrophy, and Charcot–Marie–Tooth disease [10,11].

Motion and inertial sensors embedded in different devices have been used to monitor the 6MWT performance with diverse populations [3,4,5,12]. For example, Qureshi et al. [12] examined the changes in gait speed and gait cycle length variance over six minutes and the relationships between these variables with functional systems scores, modified fatigue impact scale, and multiple sclerosis walking scale. On the other hand, Daines et al. [3] used the 6MWT to develop a smartphone sensor-based fall-risk classification method in persons with lower-limb amputations. In the same line, Drover et al. [4] developed a wearable sensor-based faller classification method for older adults using accelerometer-based features from walking and turns during the 6MWT. Therefore, according to the existing evidence, the 6MWT can help track performance changes and detect emerging medical conditions early.

Despite the increasing work, to our best knowledge, no study synthesized the available evidence regarding the monitoring and detection of emerging medical conditions during the 6MWT using motion and inertial sensors. For example, a previous systematic review examined studies that used wearable inertial sensors for the gait assessment during the 6MWT [13]. However, the study only focused its review on wearable sensors for gait assessment, and none of the articles included pediatric populations [13]. Therefore, in this systematic review, we aim to synthesize the current state-of-the-art approaches for monitoring the 6MWT performance in a wide range of clinical populations. Furthermore, we focus on technological solutions to automate the 6MWT to be performed and measured at persons’ homes without medical personnel supervision. Additionally, we analyze the benefits of its continuous measurement and discuss the potential benefits regarding recovery and early detection of emerging medical conditions.

Different medical conditions need solutions for remote treatment and monitoring. As the sensors are available in commonly used mobile devices, and various physical tests can be performed independently by the population, the automatic analysis of the data can help detect multiple diseases. Automated detection is focused on using technology with artificial intelligence techniques and statistical analysis to identify different patterns related to walking, falls, and others. The system that will be developed will promote continuous measurement with the sensors, and the medical doctors can check the progression of its medical conditions remotely. The performance of these measurements is important for recovering some diseases, e.g., stroke, early detection of diseases, e.g., Parkinson’s disease, or continuous monitoring, e.g., older adults. Other conditions can be monitored, and further development will benefit the system.

The remainder of the paper is structured as follows. First, Section 2 elaborates the methodology, presenting the research questions, search strategy, and inclusion criteria. Then, in Section 3, we show the results, offering the most relevant works and discussing them. Next, in Section 4, we discuss them from a medical point of view, and finally, Section 5 concludes the paper.

## 2. Methodology

This systematic review follows the “Preferred Reporting Items for Systematic reviews and Meta-Analyses” (PRISMA) methodological framework [14]. The goal of this methodology is to identify and gather relevant articles based on specific inclusion and exclusion criteria using well-defined search keywords, sanitize the results by removing duplicates and other irrelevant or incomplete articles from the result set, and pick the articles that should undergo thorough screening after performing a qualitative analysis on the sanitized result set. In addition, this study aims to capture and analyze the current trends in using 6MWT available in the literature. To achieve these goals, we automatically used a natural language processing (NLP) toolkit [15] to further an efficient and exhaustive search of the literature corpus. 

### 2.1. Six-Minute Walk Test Protocol

First, we want to summarize the protocol for the 6MWT so that the remainder of the article can focus on specific methods to perform these measurements. Traditionally, this test is measured with equipment, including a stopwatch, a measuring wheel to measure the distance covered, and two objects (e.g., cones) to mark the distance that needs to be covered. Optionally, a pulse oximeter can measure heart rate, SpO2, and Borg breathlessness scale. Finally, at least 30 m of the unimpeded walkway are needed (e.g., a hallway). Cones are usually placed as turning points at the end of the 30-m stretch. As a precaution, chairs are also placed at the turning points.

Patients usually receive instructions to walk back and forth around the marked turning points for 6 min. They are also informed that getting out of breath or becoming exhausted is normal, and slowing down, stopping, or resting is permitted, if necessary. Patients may lean against the wall while resting but should resume walking as soon as possible.

### 2.2. Research Questions

The main questions of this systematic review were as follows: (RQ1) Which are the most used sensors for measuring the 6MWT results? (RQ2) Which are the benefits of the measurements based on the 6MWT? (RQ3) How do the sensor measurements related to the 6MWT contribute to treating different diseases?

### 2.3. Inclusion Criteria

The 6MWT is being studied for its implementation with different sensors available. Therefore, the selection of the different studies for this systematic review was performed with the following eligibility criteria: (1) studies that measure the results of the 6MWT with sensors; (2) studies that present different analyses of six-minute walk test; (3) studies that present the purpose of the study; (4) studies that characterize the population of the study; (5) studies that present precise results about 6MWT; (6) studies presenting original research; (7) studies published between 2016 and 2021; (8) studies written in English.

### 2.4. Search Strategy

The research strategy followed a PRISMA methodology to identify and process the literature on 6MWT published from January 2016 to July 2021. Through the NLP toolkit, the following electronic databases were explored automatically for articles selection: IEEE Xplore, Springer, ScienceDirect (i.e., Elsevier), and PubMed. 

The NLP framework input parameters are a collection of keywords used to identify potentially relevant articles and a set of properties that should be satisfied by the identified articles. The following research keywords were used: “Six-Minute Walk Test” AND “sensors” AND “measurement”. The program automatically eliminated all duplicates based on the DOI numbers. Additionally, the relevant papers were identified based on the initial keyword search and the inclusion criteria. For more detailed information about the features of the NLP toolkit, we refer the reader to the study by Zdravevski et al. [15].

Every identified study was independently evaluated by the authors, determining their suitability with the eligible criteria of the search. It is multidisciplinary research that includes people for health, sports, and computer sciences. The people from computer sciences (I.M.P., H.V.D., E.Z. and J.F.M.) analyzed the data related to the sensors and how they can be used in the medical field, specifically related to 6MWT. However, 6MWT is related to different pathologies, and the analysis of the medical things was performed by medical researchers (M.V.V., J.S. and C.A.). Finally, the 6MWT is being used by sports people (D.L.M.) to measure the functionality in older adults. The studies were analyzed to identify the various methods for using sensors to measure the 6MWT results. The research was performed during July 2021.

### 2.5. Extraction of Study Characteristics

Different data were extracted from the further studies. The other collected parameters are presented in Table 1: year of publication, location, the population of the study, purpose, sensors used, presence of medical collaborators, types of methods implemented, and diseases present in the population analyzed. Unfortunately, the source code and datasets used in the analyzed studies are not publicly available. Thus, we asked the different authors about the source code and datasets without answering.

## 3. Results

**Figure 1:**
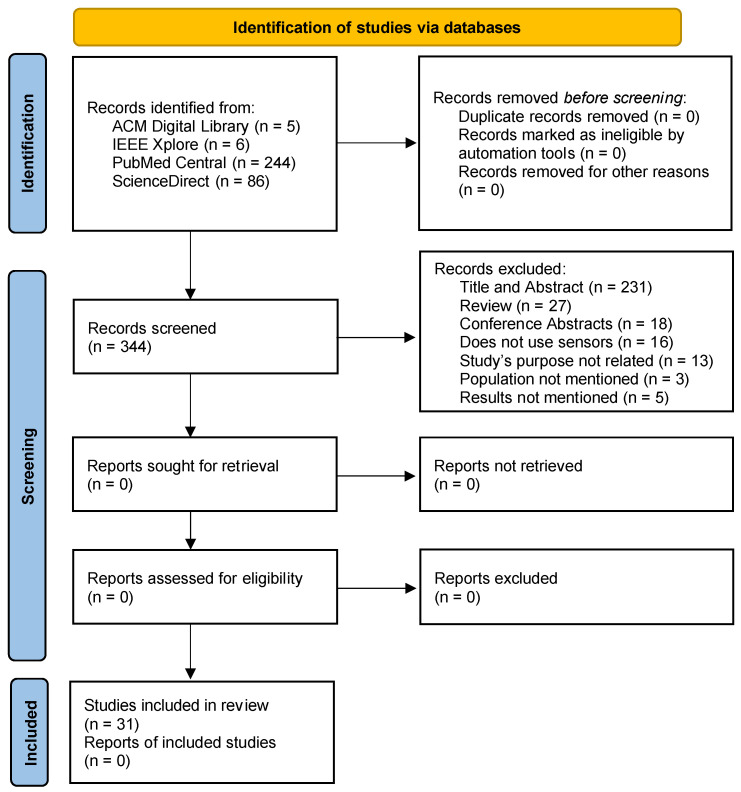
Flow diagram of the selection of the papers.

As presented in Figure 1, we identified 341 records from the selected sources. After analyzing each research article’s title and abstract, 231 papers were excluded by the unrelated 6MWT. Next, the literature reviews were excluded, resulting in the exclusion of 27 studies. Another reason for exclusion was the article type, excluding 18 conference abstracts. Then, the full text of the remaining 65 papers was analyzed, resulting in the exclusion of 16 studies that did not consider the sensors. Additionally, 13 studies were excluded because the purpose is not directly related to the 6MWT, 3 studies that did not mention the population or sample studied, and 5 studies that did not present the results. Finally, the remaining 31 research articles were examined and included in the qualitative and quantitative syntheses.

The selected studies were examined to extract the relevant data. The query performed in this study retrieved papers published between 2016 and 2021. As reported in Table 1, six studies (19%) were published in 2021, seven studies (23%) in 2020, six studies (19%) in 2019, four studies (13%) in 2018, four studies (13%) in 2017, and four studies (13%) in 2016. Regarding the location of the different studies, eight studies (26%) were performed in the USA, five studies (16%) in France, three studies (10%) in Italy, three studies (10%) in the UK, four studies (13%) in Canada, two studies (6%) in Switzerland, and the remaining studies (3%) in different countries, including Brazil, Belgium, Egypt, Australia, Israel, Korea, and Germany. Regarding the sensors used, 29 studies (94%) used motion and inertial sensors embedded in different devices, and the remaining 2 studies used Diffusion Tensor Imaging and Global Positioning System (GPS). Only four studies (13%) did not have medical collaboration. Regarding the type of methods implemented, most (97%) implemented statistical methods for the analysis, and only two studies (6%) used machine learning methods. Finally, 10 studies (32%) were related to multiple sclerosis, 6 studies (19%) to pulmonary diseases, 3 studies (10%) to heart diseases, 2 studies (6%) to brain injuries, 5 studies (16%) to bone diseases, 1 study (3%) to kidney diseases, and 3 studies (10%) did not define any disease.

The authors of [14] used wearable sensors embedded in a BeatWalk device, including ankle-worn and inertial sensors, and individualized musical stimulation for gait auto-rehabilitation with 6MWT performance. A total of 45 patients with Parkinson’s disease have gait disorders, but they walk without aids, aged 65 ± 9 years old, with moderate disease severity. The test was applied before and after the rehabilitation program, revealing that, on average, the distance walked increased by 17.63 m, the number of steps per minute increased by 3.07, the velocity increased by 0.04 m/s, the stride length increased by 0.02 m, and the asymmetry index decreased by 0.01%. The comparison was performed with Wilcoxon signed-rank test, and the whole data was also tested with Chi-squared tests or Fisher’s exact tests. The authors used musical stimulation during the experiments to improve the results on the 6MWT, where they verified that the results improved during and after the training program was applied. The 6MWT is an easy test that allows the authors to evaluate the gait auto-rehabilitation at home. The sensors were used to acquire the spatiotemporal gait parameters to control the variations during the drug cycle.

Hadouiri et al. [15] used the GAITRite walkway system (CIR Systems Inc., Franklin, NJ, USA) and the software PKMAS (ProtoKinetics, Havertown, PA, USA) for the measurement of different spatiotemporal variables during the 6MWT, including velocity, number of steps per minute, stride length, stride width, and double support time. In addition, the authors evaluated the performance of the inverted pendulum (IP) algorithm and an adaptation correcting for lateral step movement. The study consisted of the analysis of 45 patients with multiple sclerosis and 24 healthy individuals. The test was applied before and after the rehabilitation program in the two groups. Regarding the patients with multiple sclerosis, the values revealed that, on average, the distance walked increased by 19 m, the number of steps per minute increased by 3.4, the velocity increased by 0.05 m/s, the stride length increased by 0.01 m, the stride width maintained, and the double support time decreased by 0.89%. Regarding the healthy people, the values revealed that, on average, the distance walked increased by 34 m, the number of steps per minute increased by 5.58, the velocity increased by 0.11 m/s, the stride length increased by 0.03 m, the stride width maintained, and the double support time decreased by 1.2%. Additionally, the data analysis was performed with SAS version 9.4 (SAS Inc., Cary, NC, USA), measuring the mean and standard deviation of the acquired values. Finally, its homogeneity was evaluated with Kolmogorov–Smirnov and Levene’s tests. Thus, the authors accurately measured different spatiotemporal variables during 6MWT, which were used to adapt the rehabilitation care depending on the particular situation of other patients.

In [16], sensorized insoles (FlexInFit^®^, Sensormedica, Guidonia Montecelio, Rome, Italy) were used by two groups of participants with a total hip replacement, such as the experimental group composed of 19 patients and the control group formed by 21 patients to analyze the visual biofeedback effect for plantar pressure dynamic evaluation. The participants in the experimental group were, on average, 64.12 years old, with a height of 166.42 cm, and a weight of 72.74 kg. The participants in the control group were, on average, aged 61.30 years old, with a height of 172.00 cm, and a weight of 86.29 kg. The analyzed parameter related to the 6MWT was the distance. Thus, the experimental groups reported 194.3 m before the surgery and 308.4 m after the surgery. Additionally, the control group reported, on average, 187.5 m before the surgery and 310.2 m after the surgery. The data were analyzed with Shapiro–Wilk test to test the normality of the average, Pearson’s chi-squared test to test the associations between variables, Student’s *t*-test, and the analogous nonparametric Mann–Whitney U to test the different variables with the STATA statistical software version 14 (Stata Corporation, College Station, TX, USA, 2015). The authors used the 6MWT to determine the ability to walk after a total hip replacement. The sensors were used to easily measure the different distances during the rehabilitation phase and estimate the timeline to recovery.

Sagawa et al. [17] recruited 41 participants with multiple sclerosis and 16 healthy individuals as the reference group to determine the level of activity and establish associations between clinical parameters. The participants with multiple sclerosis were, on average, aged 51.3 years old, with a height of 1.67 m, and a weight of 75.4 kg, and they were mainly female persons. The healthy participants were, on average, aged 48.7 years old, with a height of 1.72 m, and a weight of 72.3 kg. The authors only measured the distance of the 6MWT, reporting an average of 267 m in participants with multiple sclerosis and 649 m in healthy participants. The Statistica version 10 (StatSoft, Tulsa, OK, USA) was used to evaluate the mean values with Student’s *t*-test, Chi-square test, one-way analysis of variance, and Tukey post-hoc tests. The 6MWT was used to measure the maximum distance walked for 6 min and the endurance of walking. The sensors were used to measure the different variables easily.

The authors of [18] used a mobile application that collects data from inertial sensors, a global positioning system (GPS) receiver, and a Bluetooth pulse oximeter for the assessment of the accuracy of the indoor 6MWT in clinical settings, the validity, and test–retest reliability of outdoor 6MWT in the community, the compliance, usability, and acceptance of the mobile application, and the feasibility of pulse oximetry during the 6MWT. For the performance of the tests, 30 individuals who participated with pulmonary hypertension were recruited, where 37% were male, and 63% were female, and their age, on average, was 50 years old. The participants used different devices, where three 3 patients used Android phones, twenty 20 used Apple iPhones, and nine 9 used both. In addition, the authors collected various features, e.g., distance. Finally, they were analyzed with statistics for the differences and Bland–Altman plots, revealing that the mobile application was sometimes inaccurate. Still, the results were correlated with the conventional application of the test. The 6MWT was used to assess the distance walked in patients with pulmonary arterial hypertension, where the sensors allow the quick computation of the walking distance in indoor and outdoor environments.

Tan et al. [19] recruited 11 patients aged between 20 and 60 years old with an average of 46 years old with spinal cord injuries. The participants used an optical motion capture system (Optotrak Certus; Northern Digital, Waterloo, ON, Canada) to track movements of infrared light-emitting rigid body sensors to examine the walking performance and intralimb motor coordination. For this purpose, the authors measured the distance. First, they performed different statistical tests with SPSS^®^ 24 statistical software (Version 22, IBM Inc., Armonk, NY, USA), including Shapiro–Wilk’s test, Levene’s test, Greenhouse–Geisser corrections, linear mixed model, repeated-measures analysis of variance (rmANOVA), and Mann–Whitney U tests. The results revealed an increased distance in the performance of the 6MWT. Next, the authors used the 6MWT to quantify the walking performance. Finally, the sensors were used to track the motion of the fifth metatarsophalangeal, ankle, knee, and hip joints to correct the test’s evaluation.

In [20], the authors investigated the presence of local dynamic stability of gait in 80 patients with multiple sclerosis with minimal impairment and 20 participants in a control group during the performance of the 6MWT at their maximum speed. The different variables were measured with inertial sensors, calculating the short-term Lyapunov’s exponents. The acquired data were tested with the Wilcoxon rank-sum test, Fisher’s exact test, and mixed-design ANOVA, revealing that the gait speed was lower in patients with multiple sclerosis, high impact of fatigue, and poor balance. The authors used the 6MWT to assess the gait speed during its performance, where the used sensors allow the easy measurement of the gait speed, promoting remote monitoring.

Gulart et al. [21] used an accelerometer sensor to determine the cut-off point for the London Chest Activity of Daily Living scale to discriminate better functional status and the scores associated with clinical outcomes of a pulmonary rehabilitation program. They analyzed 61 patients with chronic obstructive pulmonary disease who were, on average, 65.5 years old, with a height of 1.67 m, and a weight of 72.7 kg with the distance measurement during the 6MWT. The acquired data were analyzed with SPSS Statistics 20.0 software to apply the Shapiro–Wilk test and calculate the Spearman correlation coefficients. The final comparisons were performed with Mann–Whitney U test, resulting in low correlations. In addition, the authors used the 6MWT to measure the distance walked during the test, where the use of sensors allows its calculation more accurately.

The authors of [22] use the steps measured by a Mi Band 2 device to evaluate its validity and reliability during the 6MWT performance. The study was composed of 14 healthy individuals, which, on average, were 23 years old. The study consisted of measuring the distance and speed during the test for further analysis. The analysis was performed with SPSS Statistical Software (Version 21), measuring the percent errors in the right and left wrist, comparing them with sample *t*-tests, and calculating the intra-class correlation coefficients. During the test, the patients walked, on average, 528 m with a speed of 5.3 km/h. The 6MWT is an easy test that allows the measurement of the number of steps during its performance. In addition, sensors were used to assess the validity and reliability of the Mi Band 2 wearable activity monitor.

Plotnik et al. [23] used six Opal motion sensor-based gait analysis systems (APDM, Portland, OR, USA) to assess gait asymmetry and bilateral coordination of gait during the 6MWT. They recruited 92 patients with multiple sclerosis and grouped them by disease severity. During the test, the authors acquired the distance covered and gait variably, performed the repeated measures ANOVA treating each 1 min interval, and calculated the correlation coefficients for each variable. They concluded that patients with lower severity presented better results. The 6MWT allows the assessment of persons with multiple sclerosis. The commodity and accurate measurement are important for the different measurements, where the sensors may be robust for these measurements for the other patients.

In [24], the authors used motion and heart rate sensors to compare the 6MWT in 107 patients with different stages of mitral and aortic valve disease. The patients were, on average, aged 66 years old, where 38 have aortic valve diseases, and 69 have mitral valve disease. Furthermore, since 65 participants were male, multiple correlations were performed between gender and disease types. Finally, on average, the participants walked 519 m during the 6MWT. Still, only 96.71% achieved the target of the distance. The 6MWT is commonly used to measure exercise tolerance and predict patient-centered outcomes, allowing the assessment of the exercise capacity and the comparisons between patients. Furthermore, it is important to evaluate the effects of therapeutic interventions and prognosis. In addition to the sensors facilitating the measurement of the distance walked, heart rate sensors detect different events to differentiate the tachyarrhythmia and activity.

The authors of [25] used a 6-channel head coil on a 1.5 T Philips Gyroscan Intera (Philips, Best, The Netherlands) with single-shot echo-planar imaging to measure the changes in vestibulospinal tract and parietoinsular vestibular cortex, and relation to the balance between old and young healthy adults. Regarding the 6MWT, the authors measured the distance in 11 old adults and 12 young adults. The old adults were, on average, aged 63.36 years old, with a height of 1.63 m, and a weight of 62.82 kg. The young adults were, on average, aged 28.42 years old, with a height of 1.71 m, and a weight of 66.42 kg. In addition, the participants were compared with the Mann–Whitney U test and independent *t*-test, showing a significant decrease in older adults. The 6MWT was used to analyze motor control, using the sensors to measure the distance to detect the changes in different aged participants.

Zeitlberger et al. [26] used a smartphone application to assess self-measured objective functional impairment with the 6MWT in three patients with lumbar degenerative disc disease with a GPS receiver. Unfortunately, the authors did not perform statistical tests with the distance measured. Still, on average, the reported values were between 381 m and 624 m. Thus, the 6MWT easily allows the assessment of functional impairment. Furthermore, measuring the distances with sensors allows easy measurements in the home environment to monitor the diseases constantly.

In [27], the authors used two inertial sensors (Physilog^®^4, Lausanne, Switzerland) placed on each foot with velcro strips and calculated 25 gait parameters in 20 participants with lower-limb amputation. They were, on average, aged 59 years old, with a height of 1.73 m, and a weight of 74.27 kg. The authors used the R software 3.3.3 to apply ANOVA and Wilcoxon signed-rank tests with the stance, flat foot ratio, minimal toe clearance, cadence, and speed, measured during the 6MWT. As a result, the average values of the stance increased by 0.25%, the flat foot ratio decreased by 3.41%, the minimal toe clearance decreased by 4.91 mm, the cadence decreased by 0.07 steps/min, and the speed was maintained. Thus, the 6MWT was instrumental in this research to monitor the evolution of different gait parameters after amputating a lower limb. Furthermore, it allowed the medical personnel to adapt the rehabilitation protocol depending on personal parameters and recovery speed.

The authors of [28] proposed using a proprietary device named SWING wearable multi-sensor system, which integrates an accelerometer, a gyroscope, a magnetometer, and up to three time-of-flight distance sensors. It was used to create a step counter based on the direct measurement of inter-leg distance. The number of participants in the study were five women and eight men with an average age of 42 years old, an average height of 1.74 m, and an average weight of 75 kg. The measurement of the distance reported an error of 2%. Thus, the 6MWT allowed reliable and relevant measurements of inter-leg distance and accurate step counting using sensors to monitor the progression of patients with multiple sclerosis.

Camp et al. [29] proposed using the SenseWear triaxial accelerometer to assess convergent, discriminant, known-group validity and floor/ceiling effects of the de Morton Mobility Index with the performance of the 6MWT. The 22 participants with acute exacerbation of chronic obstructive pulmonary disease had an average age of 60 years old, mainly had smoking habits and walk with aids. The sensors reported the distance and gait velocity during the test, and the data were analyzed with the SAS 9.4 (SAS Institute, Cary, NC, USA) to estimate the Spearmen’s correlations. The results reported a moderate positive correlation (i.e., correlation coefficient equals 0.61), which showed moderate to strong validity of the implemented method. In this research, the 6MWT was a feasible metric to perform mobility assessment in hospitalized patients with an acute exacerbation of chronic obstructive pulmonary disease. Similar to other research, the different measured parameters with sensors allowed the identification of distinct patterns in the disease progression and the recovery process.

In [30], the authors used three devices, including D-Jogger (Sennheiser, Hamburg, Germany), two iPod devices (Apple, California, USA), and three OPAL wearable sensors (Mobility Lab, APDM, Portland, OR, USA) to measure the spatiotemporal gait parameters and establish the comparison between patients with multiple sclerosis and healthy people. They recruited 31 patients with multiple sclerosis with an average age of 53.45 years old, an average height of 1.71 m, and an average weight of 69.10 kg. Additionally, 30 healthy individuals were recruited with an average age of 51.77 years old, an average height of 1.70 m, and an average weight of 71.15 kg. During the 6MWT performance, the distance was measured and further analyzed with Shapiro–Wilk test, ANOVA test, Student *t*-test, and Turkey’s test with the SAS JMP Pro 13.2.0 (SAS Institute Inc., Raleigh, NC, USA). The results reported an average distance of 377.56 m in patients with multiple sclerosis and 559.46 m in healthy people. The authors measured the parameters with this test and correlated them with perceived fatigue. Thus, it allowed the establishment of some patterns about how different parameters can help identify patients’ fatigue with multiple sclerosis. Moreover, the authors established a variability of these parameters about whether the test was performed with a metronome, in silence, or to music.

The authors of [31] recruited nine participants with relapsing-remitting multiple sclerosis with an average age of 45 years old and 26 healthy individuals with an average age of 45 years old. They performed the 6MWT equipped with seven wireless inertial sensors MIMUs (Xsens MTw, Xsens Technologies, Enschede, The Netherlands). Each one was placed on the pelvis and thigh, shank, and foot of both lower limbs to measure the changes in gait kinematics due to fatigue. The features extracted from the acquired data are the range of motion related to hip, knee, and ankle joint, tested with the Shapiro–Wilk test, and Student *t*-test. The reported results discovered significant effects of walking-related fatigue on gait kinematics with reducing the range of motion during the performance of 6MWT.

Tousignant et al. [32] used biomedical sensors wirelessly transmitting a real-time ECG signal (180° eMotion Faros device), oxygen saturation, and heart rate (Nonin WristOx2 3150 device) to evaluate its feasibility in telerehabilitation based on 6MWT. Four male participants with heart failure disease were recruited with an average age of 66.25 years old. However, only statistical measurements have been performed, verifying that the mean variation of walking distance was 44 m. Thus, even though this paper illustrates that sensors are beneficial for obtaining reliable measurements, it did not systematically and thoroughly analyze the direct benefits of the 6MWT.

The authors of [33] researched the relationship between lower limb muscle activation patterns and chronic gait deficits in individuals who previously experienced a traumatic brain injury with accelerometer and electromyography sensors. The sensors measured different parameters, including the distance and walking speed during the 6MWT performance. The study included 44 individuals after traumatic brain injury, which had, on average, 53.4 years old, and 28 were females. Additionally, 20 healthy control subjects, which had, on average, 25.3 years old, and 10 were females, were included. The acquired data was tested with SPSS Statistical Software (v. 24, IBM Corp., Armonk, NY, USA), implementing the Mann–Whitney U tests, Shapiro–Wilk’s test, and Spearman’s rank-order correlation. The results reported that the average distance in the experimental group was 386 m, whereas the standard distance in healthy subjects was between 400 m and 700 m. Additionally, the results were not correlated to walking speeds. Performing such an analysis was only possible because of using systematic sensory measurements.

In [34], the authors used inertial sensors (RehaGait) capturing three-dimensional foot accelerations to evaluate the acceleration gait and the exercise-induced changes in patients with symptomatic lumbar spinal stenosis. The 6MWT was performed by 24 healthy individuals, where 9 were males and 15 were female, and they were, on average, 59.9 years old, with an average height of 1.69 m, and an average weight of 68.5 kg. Additionally, the test included 19 patients with symptomatic lumbar spinal stenosis, where 11 were males and 8 were female, and they were, on average, 73.8 years old, with an average height of 1.68 m, and an average weight of 75.8 kg. The data were analyzed with SPSS Version 21 (IBM Corporation, Armonk, NY, USA), measuring the results of the Shapiro–Wilk test, the Mann–Whitney U test, the ANOVA test, and the Bonferroni post-hoc tests. The average distance measured with healthy individuals was 410.7 m, and the value of the patients with symptomatic lumbar spinal stenosis was 361.4 m. Thus, the 6MWT and the inertial sensor-based measurements allowed the authors to perform statistically significant analysis and comparison of healthy and patients with symptomatic lumbar spinal stenosis.

D’Alessando et al. [35] assessed the prevalence and correlation of sarcopenia among elderly male patients with chronic kidney disease based on SenseWear Armband (SWA, BodyMedia, Inc., Pittsburgh, PA, USA) for the measurement of the distance walked during 6MWT. The participants included 80 patients over the age of 60 with an average weight of 80.6 kg, 40 patients aged 75 or over with an average weight of 77.3 kg, and 40 patients aged between 60 and 74 with an average weight of 84 kg. The distance walked during 6MWT was analyzed with SPSS v.25 (SPSS Inc., Chicago, IL, USA), performing descriptive analysis. The reported average values were 282 m for the patients aged 75 or over and 336 m for those between 60 and 74 years old. The authors identified that the average daily physical activity was lower in the older seniors than younger ones based on the performed measurements. Furthermore, among older seniors, sarcopenic and non-sarcopenic ones differed in age and performance on the 6MWT.

The authors of [36] also used the GAITRite walkway system (CIR Systems Inc., Franklin, NJ, USA) for the measurement of the effect of vascularized fibula free flap (VFFF) harvesting on spatiotemporal gait variables, including distance, cadence, velocity, stride length, stance, and step length, during the 6MWT. The participants in the study were 11 patients with vascularized fibula free flap and 11 healthy individuals. The patients with vascularized fibula free flap were, on average, 59 years old, with an average height of 1.70 m, and an average weight of 66 kg. The healthy individuals were, on average, 53 years old with an average height of 1.72 m and an average weight of 72 kg. The analysis of the acquired data was performed with Statistica version 10 software (Stat-Soft, Inc., Tulsa, OK, USA), measuring the results with descriptive statistics, implementing Mann–Whitney U test, ANOVA test, and Wilcoxon signed-rank test. The average results revealed that the distance was 436 m in patients with vascularized fibula free flap and 632 m in healthy individuals. Next, the velocity along the test decreased 0.1 m/s in patients with vascularized fibula free flap and 0.01 m/s in healthy individuals. Sequentially, the stride length along the test decreased 0.02 m in patients with vascularized fibula free flap and 0.04 m in healthy individuals. Next, the cadence along the test decreased 2.2 steps per minute in patients with vascularized fibula free flap and 4.0 steps per minute in healthy individuals. Sequentially, the stance along the test increased 0.02% in patients with vascularized fibula free flap and 0.35% in healthy individuals. Finally, the step length along the test was maintained in patients with vascularized fibula free flap and decreased 0.01 m in healthy individuals. With the help of the 6MWT, the authors were able to identify a significantly lower velocity between the beginning and end periods only for the VFFF group, suggesting an alteration in physical management. In conclusion, these results indicate that VFFF harvesting could alter gait and joint integrity.

In [37], the authors used spirometry to assess the metabolic disturbance and its reflection on the muscular state in interstitial pulmonary fibrosis patients. Three groups of 22 patients were analyzed, including normal people with an average of 48.9 years old, interstitial pulmonary fibrosis patients with an average age of 55.8 years old, and another group with interstitial pulmonary fibrosis patients with an average age of 51.1 years old. The data were analyzed with SPSS Statistical Software v22 (SPSS Inc., Chicago, IL, USA) for the performance of descriptive statistics, Chi-square or Fisher exact tested proportion independence, ANOVA test, paired *t*-test, and Mann–Whitney tests. The tests revealed that the average distance during the performance of 6MWT was higher (+148.4 m) in patients without oxygen therapy.

Kennedy et al. [5] also used the GAITRite walkway system (CIR Systems Inc., Franklin, NJ, USA) to research changes in spatiotemporal gait parameters and functional ambulation with several assessments, including the 6MWT. In the study, 27 children with Charcot–Marie–Tooth disease performed the assessments, in which 18 were males, the average age was 12.2 years old, the average height was 1.52 m, and the average weight was 47.1 kg. In addition, the authors performed initial assessments and another set of evaluations after 12 months, where the distance during the test was on the parameters measured. The acquired data were analyzed with the Stata (version 14) software package, descriptive statistics, and Shapiro–Wilk test results. The analysis revealed that the results slightly decreased, i.e., the average distance decreased 1 m in 12 months. The different parameters measured by the sensors during the 6MWT were instrumental in this analysis for an objective comparison between the other groups.

The authors of [38] used BioStampRC devices (MC10, Inc., Lexington, MA, USA), MTx inertial sensors (Xsens Technologies B.V., Overissel, The Netherlands), and tri-axial activity tracker GT3X (Actigraph, Pensacola, FL, USA). The sensors were used to measure gait characteristics, including the distance walked during 6MWT, the number of steps, the stride time, the swing time, and the step time of 45 patients with multiple sclerosis and 15 healthy subjects under controlled conditions. The measurements were analyzed with MATLAB code (The MathWorks, Natick, MA, USA) and IBM SPSS Statistics for Windows (version 22; IBM SPSS Inc., Armonk, NY, USA). The software was used to perform descriptive statistics, Bland–Altman plots, ANOVA analysis, and Mann–Whitney U test for post-hoc analysis. The average distance and the average step number decreased with the severity of the disease. On the other hand, the average stride time, the average swing time, and the average step time increased with the severity of the disease.

In [39], low-cost motion sensors were used to assess the functional capacity in older adults with chronic obstructive pulmonary disease. For this purpose, the 6MWT was applied, and the distance was measured. The sample in the study consisted of 49 individuals, where 30 were males and 19 were female. Firstly, the male subjects were, on average, aged 72 years old, with an average weight of 86.5 kg, and an average height of 1.75 m. Secondly, the female subjects were, on average, aged 71 years old, with an average weight of 71.4 kg, and an average height of 1.59 m. Finally, the whole sample was aged 72 years old, with an average weight of 80.7 kg, and an average height of 1.69 m. The data analysis was performed with JMP version 11 (SAS Institute, Inc., Cary, NC, USA), measuring the different correlations. They also used the multivariable linear regression model, Shapiro–Wilk test, and paired *t*-test to obtain the results. The results showed a higher average distance in male subjects than female subjects based on the measurements during the 6MWT.

Cheng et al. [40] used the accelerometer and spirometry sensors to create classification models to measure the pulmonary function in 24 patients. The features were extracted with a feature selection approach, where one of them was the cadence. The patients were, on average, aged 76 years old, with an average height of 1.67 m, an average weight of 83 kg, and only nine were female. The data were analyzed with support vector machine (SVM) models, reporting that the patients may use their mobile phones to support automatic computation of pulmonary function. In addition, the accelerometer and spirometer sensors provided reliable time-series data that contained patterns related to the different conditions of the patients.

The authors of [41] used only the accelerometer sensor to implement several models to monitor cardiopulmonary conditions, and they created a new model named DPClass. The study included 55 pulmonary patients and 11 healthy subjects to measure the cadence and velocity with the sensors’ data. Thus, the authors implemented linear and radial basis function (RBF)—SVM models, decision trees, and the DPClass model with pattern classification techniques. The overall results reported that the best accuracy was achieved with the RBF SVM model. The second one was the DPClass model, proving the reliability of the implemented model. Ultimately, the whole approach was reliable on the accelerometer measurements to facilitate the corresponding analysis of the cardiopulmonary conditions.

In [42], the GAITRite walkway system (CIR Systems Inc., Franklin, NJ, USA) was also used to establish the relationship between the walk ratio and other measurements in 229 patients with multiple sclerosis. On average, the patients were aged 43.4 years old, with a height of 1.68 m, a weight of 69.4 kg, and the gender distribution consisted of 143 females and 86 males. The authors acquired the distance during the 6MWT. It was also compared with descriptive statistics, correlation coefficients, and ANOVA tests with SPSS software (Version 23.0 for Windows, SPSS Inc., Chicago, IL, USA). The results reported an average distance of 423.4 m with a low correlation with walk ratio. Thus, the measure of gait control through the walk ratio was facilitated by the 6MWT measurements of patients with multiple sclerosis.

Qureshi et al. [10], a blockchain-based service network (BSN) platform called TEMPO3.1 analyzes changes in gait speed and gait cycle length variance during 6MWT, determining the relationships between these variables and functional systems scores, modified fatigue impact scale, and multiple sclerosis walking scale. The study recruited 28 patients with multiple sclerosis aged between 18 and 65 years old. The results reported that fatigue was high in the analyzed patients, and it reported high variance in the gait cycle length in the last minute of the test.

## 4. Discussion

### 4.1. Interpretation of the Results

An increase in the distance walked indicates improvement in essential mobility. Resnik et al. [45] suggested that in amputee rehabilitation and post-training, a difference of at least 45 m should be observed for the 6MWT to ensure a tangible change in the patient’s condition.

In some neuromuscular conditions, such as Duchenne/Becker muscular dystrophy, spinal muscular atrophy, Charcot–Marie–Tooth disease, and myasthenia gravis, the 6MWT has been implemented in the assessment of those patients. Additionally, it has been regularly used to capture any changes, which provide valuable information regarding the natural history of these disorders.

Some studies used the 6MWT to identify patients with neuromuscular junction dysfunction [46]. By splitting the test into six different components where each minute is an additional data point, the researchers discovered that the test could identify any malfunctions in the neuromuscular junctions. Likewise, comparing the distance covered in the first and the last minute suggested how much the fatigue had influenced the individual.

**Figure 2:**
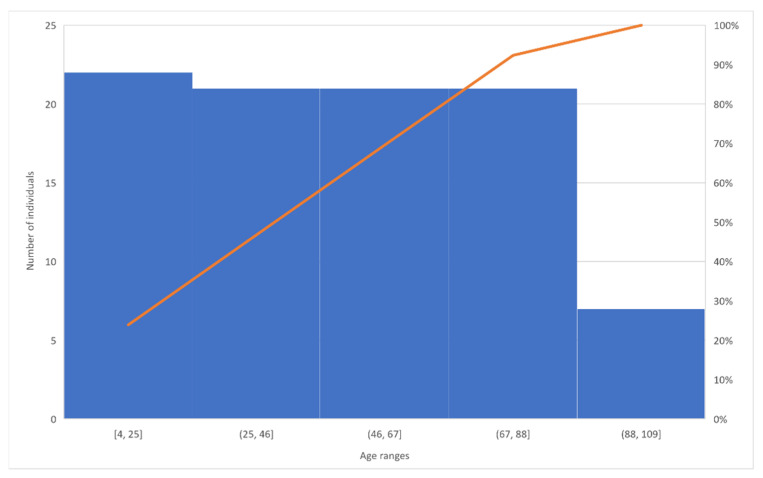
Relation between age ranges and number of individuals.

As presented in Figure 2, after analyzing the 31 studies, we verified that more than 90% of the studies were focused on individuals aged more than 67 years old. Additionally, more than 65% of the studies were focused on individuals aged more than 46 years old.

**Figure 3:**
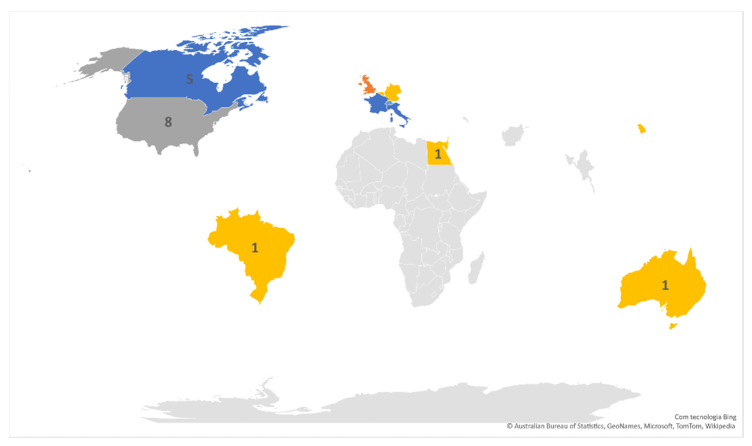
Relation between the number of studies and countries.

Regarding the countries of the studies selected according to our inclusion criteria, presented in Figure 3, the higher ratio of eight studies (23%) was performed in the United States of America, five studies for each country (14%) were conducted in Italy, France, and Canada, and the remaining countries do not have more than three studies.

**Figure 4:**
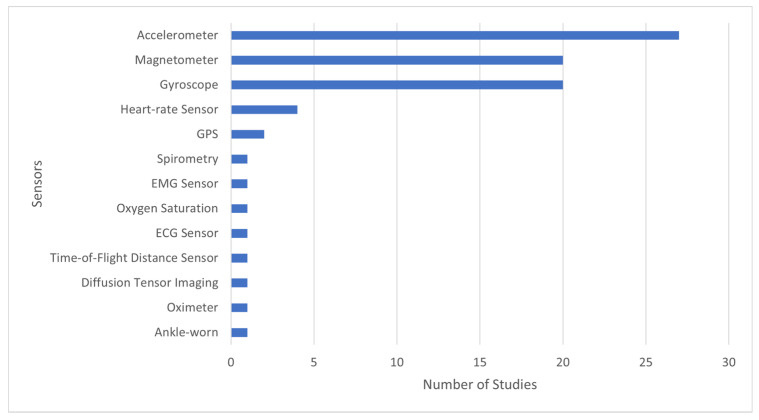
Relation between sensors and number of studies.

Regarding the sensors used in the various studies presented in Figure 4, 33% considered accelerometer sensor, 25% applied the magnetometer and gyroscope, and 5% used the heart-rate sensor. In addition, other residual sensors were used, including GPS receiver, spirometry, EMG sensors, oxygen saturation, ankle-worn, oximeter, diffusion tensor imaging, time-of-flight distance sensor and ECG sensor.

**Figure 5:**
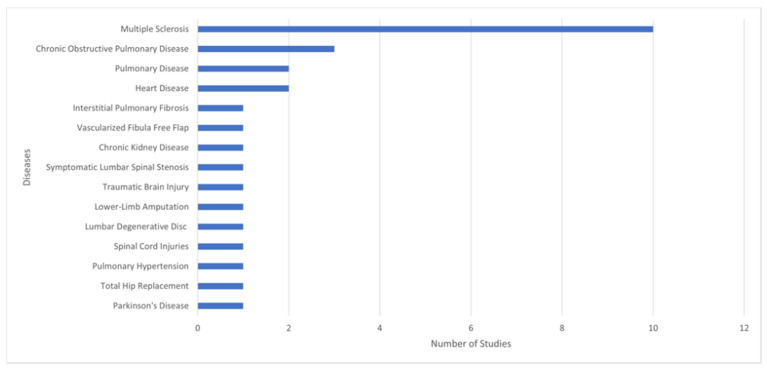
Relation between diseases and number of studies.

Regarding the diseases studied in the various papers presented in Figure 5, 36% considered multiple sclerosis, 11% applied the chronic obstructive pulmonary disease, 14% used the heart (7%) and pulmonary (7%) diseases, and the other conditions were residual.

### 4.2. Validity and Reliability

Few studies included in this review analyzed the validity and reliability of wearable devices to measure the 6MWT performance [20,24]. For example, Salvi et al. [20] compared the distance measured by an app during the 6MWT with a physiologists’ app in 30 patients with pulmonary hypertension. Regarding the validity, although the authors observed high correlations (*r* = 0.89; *p* < 0.001) between both devices, the Bland–Altman plots revealed high differences because the standard deviation of the differences was 47 m [20]. According to the authors, these differences might be explained by the app’s inaccuracy, day-to-day walking performance variability, and tests incorrectly executed by not following instructions [20].

Regarding the reliability, although they found high intra-class correlation coefficients (ICC = 0.91) between both apps, the coefficient of variation and standard error of measurement was high (CV = 12.45%; SEM = 36.97 m) [20]. These authors indicated that the measurement error could be attributed to the instrument’s inaccuracy [20]. In the study by Paradiso et al. [24], the authors compared the number of steps estimated by the Mi Band 2 with those determined by hand using a video camera during the 6MWT in 14 healthy adults. The authors did not observe significant differences between results, and the Bland–Altman plots did not show a systematic error. However, they did not analyze the reliability (e.g., using the ICC, SEM, and CV analyses) of the Mi Band 2 by comparing the results with those determined by hand. The authors only presented the ICC value for the Mi Bands worn on the left vs. right wrists during the 6MWT, which was interpreted as excellent (ICC = 0.83) [20]. 

The results indicate that further research should be conducted to compare the validity and reliability of wearable devices vs. criterion reference devices to measure the 6MWT performance.

### 4.3. Comparison of the Different Studies Analyzed

For the comparison of the studies, Table 2 presents the relation between the sensors used and diseases used, verifying that more studies are needed relating to multiple sclerosis to improve the results of the other authors. In addition, a system must be developed with the combination of the different methods to accomplish the goals of monitoring people with various diseases using the 6MWT. Currently, multiple sensors are used and multiple conditions are studied, but a standard must be defined.

As presented in Table 3, different modules or systems are shown regarding the results and benefits of the studies, but the various authors are thinking about multiple purposes. Therefore, a new method or system combining the different recent studies is urged.

Based on the analysis of Table 2 and Table 3, we are proposing the use of inertial sensors to measure the results of the 6MWT, providing the results to the healthcare professionals and patients without significant intervention. Currently, the research studies proved that it could be possible to implement with the analysis of the different gait parameters.

There are no automatic systems in the market that allow the measurement of the results of the 6MWT. However, this test is essential to be measured accurately, and it can enable the creation of patient empowerment solutions. Therefore, the walking signals must be better analyzed to create a reliable and usable system.

### 4.4. Final Remarks

This systematic review proved that the development of an automatic solution for measuring the results of physical ability during the performance of 6MWT is possible. Our idea is to develop an integrated system for remotely monitoring and prescription of treatments. In 2020, the COVID-19 pandemic started, and some of the other diseases were not correctly accompanied by medical people. An urgent and reliable solution for measuring different physical disorders is essential to improve the life quality of different people. The isolation and lack of mobility of various people related to the pandemic time increase other health problems. The development of a remote system removes these barriers and returns people’s quality to normality. For these developments, different sensors, microphones, and cameras can monitor and communicate with the patients. Additionally, inertial sensors are commonly available in a mobile device to check some health diseases. The key to different treatments is communication and remote measurements.

The main findings from the 31 studies identified by this review are as follows. Concerning RQ1, “Which are the most used sensors for measuring the 6MWT results?” we conclude that the most used sensors are inertial and magnetic sensors, including accelerometers, magnetometers, and gyroscopes. Those sensors embedded in mobile devices mainly offer significant benefits because no special medical equipment is required, making the methodology and benefits available to a broader audience.

Regarding RQ2, “Which are the benefits of the measurements based on the 6MWT?”, the combination of sensors with the 6MWT allows the easy measurement of different variables to estimate various parameters related to the test. Another benefit is that the continuous measurements would provide physicians a timeline of the measured parameters offering two-fold benefit. Firstly, in the case of healthy patients, the constant measurement would provide a baseline, and any significant deviations from that could trigger additional investigations and tests. Secondly, for patients with a known medical condition, such continuous measurements would allow medical personnel to establish a pattern between stages of the disease, pre-conditions, age, and other relevant factors and the 6MWT results. Therefore, using this data, detecting the medical conditions based on the parameters of the 6MWT would be possible in the future.

Finally, related to RQ3, “How the sensor measurements related to the 6MWT contribute for the treatment of different diseases?”, we identified that the following diseases are frequently studied and monitored with the 6MWT: arthritis, fibromyalgia, geriatrics, multiple sclerosis, Parkinson’s disease, spinal cord injury, stroke, muscle disorders, spinal muscular atrophy, and Charcot–Marie–Tooth disease, among others. Still, even though there are substantial potential benefits, currently, there are no studies regarding automated methods for continuous measurement of 6MWT and these medical conditions, nor in-depth analysis of the electronic medical records and the correlation of the 6MWT results.

If electronic medical records are introduced into the analysis, undoubtedly, this will open other challenges related to data processing. First, it would present multi-modal and nominal (i.e., categorical) data that needs to be converted into numeric data so some machine learning algorithms can be applied [47]. Likewise, big data architectures become necessary for efficient and timely processing [48]. It entails using efficient algorithms for cluster size and cost optimization [49], and scalable feature selection and dimensionality reduction [50].

## 5. Conclusions

This article has performed a systematic review on sensors and automated approaches for measuring the 6MWT. A total of 31 studies were considered relevant for the inclusion criteria, which means this area is an attractive research field. In line with this, mobile devices and applications allow the 6MWT performance measurement. Furthermore, specialized mobile applications may be developed to self-assess their walking performance and report this to their physicians.

This review identified the current research trends regarding continuous applied measurements of the 6MWT, the most used sensors, and which methods yield state-of-the-art predictive and analytical performance. The information provided in this review can aid in developing systems for the automatic measurement of the results of the 6MWT. In addition, it would be vital for remote monitoring of different patients, both in telemedicine applications from the clinicians’ perspective, and providing a tool to patients to better control their health and underlying medical conditions.

## Figures and Tables

**Table 1:** Study analysis.

Paper	Year	Location	Population	Purpose of Study	Sensors Used	Include Medical Collaboration	Type of Methods	Diseases
De Cock et al. [16]	2021	Montpellier (France)	45 Parkinson’s disease patients	Investigating the observance, safety, tolerance, usability, and enjoyment of a smartphone application	Ankle-worn, accelerometer, gyroscope, and magnetometer sensors	Yes	Statistical	Parkinson’s disease
Hadouiri et al. [17]	2021	Besançon (France)	45 multiple sclerosis patients and 24 healthy subjects	Measuring the performance of the inverted pendulum algorithm, and an adaptation correcting for lateral step movement	GAITRite walkway system composed of accelerometer, gyroscope, and magnetometer sensors	Yes	Statistical	multiple sclerosis
Marin et al. [18]	2021	Pavia (Italy)	19 Total Hip Replacement and 21 healthy patients	Investigating the visual biofeedback effect of a sensorized system for plantar pressure dynamic evaluation	sensorized insoles composed of accelerometer, gyroscope, and magnetometer sensors	Yes	Statistical	Total Hip Replacement
Sagawa et al. [19]	2021	Besançon (France)	41 multiple sclerosis patients and 16 healthy subjects	Measuring the level of activity during weekdays and weekends, and its correlation with clinical parameters	accelerometer	Yes	Statistical	multiple sclerosis
Salvi et al. [20]	2021	Oxford (United Kingdom)	33 pulmonary hypertension patients	Assessing the accuracy of the indoor clinical settings, validity, and test-retest reliability of outdoor performance in the community, compliance, usability, and acceptance of the mobile application, and feasibility of pulse oximetry	accelerometer, gyroscope, magnetometer, Global Positioning System (GPS), and Bluetooth pulse oximeter	Yes	Statistical	pulmonary hypertension
Tan et al. [21]	2021	Atlanta (United States of America)	11 spinal cord injuries patients	Analyzing the daily acute intermittent hypoxia combined with walking practice	optical motion capture system composed by accelerometer, gyroscope, and magnetometer	Yes	Statistical	spinal cord injuries
Carommi et al. [22]	2020	Milan (Italy), Moncrivello—Vercelli (Italy), and Genova (Italy)	80 multiple sclerosis patients with no clinal evidence	Researching the presence of poor Local Dynamic Stability of gait	accelerometer, gyroscope, and magnetometer	Yes	Statistical	multiple sclerosis
Gulart et al. [23]	2020	Santa Catarina (Brazil)	61 chronic obstructive pulmonary disease patients	Determining the cut-off point for the London Chest Activity of Daily Living scale and the scores associated with clinical outcomes of a pulmonary rehabilitation program	Accelerometer	Yes	Statistical	chronic obstructive pulmonary disease
Paradiso et al. [24]	2020	Victoria (Canada)	14 individuals	Evaluating the measurement of steps and the assessment of the validity and reliability of the monitor to measure heart rate during rest and exercise	Mi Band device composed by accelerometer and heart rate sensors	No	Statistical	N/D
Plotnik et al. [25]	2020	Washington (United States of America)	92 multiple sclerosis patients	Assessing gait asymmetry and bilateral coordination of gait (BCG), and their association with disease severity	Opal motion sensor-based gait analysis system composed by accelerometer, gyroscope, and magnetometer	Yes	Statistical	multiple sclerosis
Schubert et al. [26]	2020	Berlin (Germany) and Sheffield (United Kingdom)	107 heart disease patients	Measuring the distances in patients with different stages of mitral and aortic valve disease	accelerometer, gyroscope, magnetometer, and heart rate sensors	Yes	Statistical	heart disease
Yeo et al. [27]	2020	Daegu (Korea)	11 older adults and 12 young people	Investigating the changes of the vestibulospinal tract, and parietoinsular vestibular cortex	Diffusion Tensor Imaging	Yes	Statistical	N/D
Zeitlberger et al. [28]	2020	St. Gallen (Switzerland)	3 lumbar degenerative disc disease patients	Assessment of the discrimination between Epidural steroid injection responders and non-responders	Global Positioning System (GPS)	Yes	Statistical	lumbar degenerative disc disease
Beausoleil et al. [29]	2019	Quebec (Canada)	15 lower-limb amputation patients	Quantification of the evolution of gait parameters	accelerometer, gyroscope, magnetometer, and heart rate sensors	Yes	Statistical	lower-limb amputation
Bertuletti et al. [30]	2019	Sheffield (United Kingdom)	13 multiple sclerosis patients	Testing and validation of a step counter method based on the direct measurement of inter-leg distance	SWING wearable multi-sensor system composed of accelerometer, gyroscope, magnetometer, and time-of-flight distance sensors	No	Statistical	multiple sclerosis
Camp et al. [31]	2019	Vancouver (Canada)	22 acute exacerbation of chronic obstructive pulmonary disease patients	Assessing convergent, discriminant, and known-group validity and floor/ceiling effects of the de Morton Mobility Index	accelerometer	Yes	Statistical	chronic obstructive pulmonary disease
Moumdjian et al. [32]	2019	Hasselt (Belgium)	31 multiple sclerosis patients and 30 healthy subjects	Investigating the effects on perceived physical and cognitive fatigue, motivation, and gait	OPAL wearable sensors are composed of an accelerometer, gyroscope, and magnetometer	Yes	Statistical	multiple sclerosis
Taborri et al. [33]	2019	Crema (Italy)	9 multiple sclerosis patients and 26 healthy subjects	Measuring changes in gait kinematics due to fatigue	wireless inertial sensors MIMUs composed by accelerometer, gyroscope, and magnetometer	Yes	Statistical	multiple sclerosis
Tousignant et al. [34]	2019	Quebec (Canada)	4 heart failure patients	Measuring the feasibility and usefulness of biomedical sensors in telerehabilitation	Electrocardiography (ECG) signal, oxygen saturation, and heart rate	Yes	Statistical	heart disease
Acuña et al. [35]	2018	Wisconsin–Madison (United States of America)	44 traumatic brain injury patients	Assessment of the relationship between lower limb muscle activation patterns and chronic gait deficits	Accelerometer and Electromyography (EMG) sensors	Yes	Statistical	traumatic brain injury
Byrnes et al. [36]	2018	Basel (Switzerland)	24 healthy individuals and 19 Symptomatic Lumbar Spinal Stenosis patients	Assessing the effects of the attractor for acceleration gait data, the exercise-induced changes in the attractor in patients with symptomatic lumbar spinal stenosis, and the exercise-induced changes in the attractor after surgical treatment.	accelerometer, gyroscope, and magnetometer	Yes	Statistical	Symptomatic Lumbar Spinal Stenosis
D’Alessando et al. [37]	2018	Le Mans (France)	40 chronic kidney disease patients	Assessment of the prevalence and correlation of sarcopenia	accelerometer, gyroscope, and magnetometer sensors	Yes	Statistical	chronic kidney disease
Hadouiri et al. [38]	2018	Besançon (France)	11 vascularized fibula free flap patients and 30 healthy subjects	Determination of the effect of vascularized fibula free flap harvesting on temporal-spatial gait variables	GAITRite walkway system composed of accelerometer, gyroscope, and magnetometer sensors	Yes	Statistical	vascularized fibula free flap
El Hosainy et al. [39]	2017	Cairo (Egypt)	22 healthy individuals and two groups of 22 interstitial pulmonary fibrosis patients	Assessment of the metabolic disturbance and its reflection on the muscular state	spirometry	Yes	Statistical	interstitial pulmonary fibrosis
Kennedy et al. [5]	2017	Parkville (Australia)	27 children	Investigating the change in spatiotemporal gait parameters and functional ambulation	GAITRite walkway system composed of accelerometer, gyroscope, and magnetometer sensors	No	Statistical	N/D
Moon et al. [40]	2017	Illinois (United States of America)	45 multiple sclerosis patients and 15 healthy subjects	Examining gait characteristics under controlled conditions remotely	accelerometer, gyroscope, and magnetometer sensors	No	Statistical	multiple sclerosis
Toosizadeh et al. [41]	2017	Tucson (United States of America)	49 chronic obstructive pulmonary disease patients	Evaluating an upper-extremity function test to assess functional capacity	accelerometer, gyroscope, and magnetometer sensors	Yes	Statistical	chronic obstructive pulmonary disease
Cheng et al. [42]	2016	Illinois (United States of America)	24 pulmonary disease patients	Measuring pulmonary functions	accelerometer	Yes	Machine Learning	pulmonary disease
Cheng et al. [43]	2016	Illinois (United States of America)	55 pulmonary patients and 11 healthy subjects	Tracking cardiopulmonary conditions	accelerometer	Yes	Machine Learning	pulmonary disease
Kalron [44]	2016	Tel Hashomer (Israel)	229 multiple sclerosis patients	Measuring the relationship between the walk ratio and other theoretically related constructs	GAITRite walkway system composed of accelerometer, gyroscope, and magnetometer sensors	Yes	Statistical	multiple sclerosis
Qureshi et al. [12]	2016	Virginia (United States of America)	28 multiple sclerosis patients	Inferring changes in gait speed and gait cycle length variance over six minutes, and the relationships between these variables and other assessments, including functional systems scores, modified fatigue impact scale, and multiple sclerosis walking scale	BSN platform composed by accelerometer, gyroscope, and magnetometer	No	Statistical	multiple sclerosis

**Table 2:** Relation between diseases and sensors used.

Study	Sensors													Diseases														
	Ankle-Worn	Accelerometer	Gyroscope	Magnetometer	GPS	Bluetooth Pulse Oximeter	Heart rate	Diffusion Tensor Imaging	Time-of-Flight Distance	ECG	Oxygen Saturation	EMG	Spirometry	Parkinson	Multiple Sclerosis	Total Hip Replacement	Pulmonary Diseases	Spinal Cord Injuries	Chronic Obstructive Pulmonary	Lumbar Degenerative disc	Lower-limb Amputation	Traumatic Brain Injury	Symptomatic Lumbar Spinal Stenosis	Chronic Kidney Disease	Vascularized Fibula Free Flap	Charcot–Marie–Tooth	Heart Disease	N/D
De Cock et al. [16]	x	x	x	x										x														
Hadouiri et al. [17]		x	x	x											x													
Marin et al. [18]		x	x	x												x												
Sagawa et al. [19]		x													x													
Salvi et al. [20]		x	x	x	x	x											x											
Tan et al. [21]		x	x	x														x										
Carommi et al. [22]		x	x	x											x													
Gulart et al. [23]		x																	x									
Paradiso et al. [24]		x					x																					x
Plotnik et al. [25]		x	x	x											x													
Schubert et al. [26]		x	x	x			x																				x	
Yeo et al. [27]								x																				x
Zeitlberger et al. [28]					x															x								
Beausoleil et al. [29]		x	x	x			x														x							
Bertuletti et al. [30]		x	x	x					x						x													
Camp et al. [31]		x																	x									
Moumdjian et al. [32]		x	x	x											x													
Taborri et al. [33]		x	x	x											x													
Tousignant et al. [34]							x			x	x																x	
Acuña et al. [35]		x										x										x						
Byrnes et al. [36]		x	x	x																			x					
D’Alessando et al. [37]		x	x	x																				x				
Hadouiri et al. [38]		x	x																						x			
El Hosainy et al. [39]													x				x											
Kennedy et al. [5]		x	x	x																							x	
Moon et al. [40]		x	x	x											x													
Toosizadeh et al. [41]		x	x	x															x									
Cheng et al. [42]		x															x											
Cheng et al. [43]		x															x											
Kalron [44]		x	x	x											x													
Qureshi et al. [12]		x	x	x											x													

**Table 3:** Study results and benefits.

Study	Results and Benefits	Limitations
De Cock et al. [16]	Results demonstrated that musical application (BeatWalk) for individualized gait rehabilitation in Parkinson’s is a safe, user-friendly, and enjoyable solution displaying good observance. The rehabilitation program using BeatWalk motivated patients to go outside, walk alone, and enjoy physical activity for almost eight and a half hours.	N/D
Hadouiri et al. [17]	The results demonstrate that Spatio-temporal (ST) variables analyzed during specific 6MWT intervals are reliable for assessing the physical management in multiple sclerosis. This process could be used in clinical practice or medical treatments during the intervals of a 6MWT.	The measurement of ST variables during the 6MWT was not continuous and depended on the length of the GAITRite instrumented walkway. Therefore, only ST variables were used to assess walking in this study, and only two tests were performed.
Marin et al. [18]	The results demonstrated improved weight-bearing distribution for the experimental group compared to the control group. This process may facilitate the performance of the rehabilitation activities magnifying the peripherical nervous system caption.	N/D
Sagawa et al. [19]	The results showed that, during the week, multiple sclerosis (PwMS) performed less activity than their healthy peers. In addition, multiple Sclerosis had a stable level of activity throughout the week, contrary to healthy persons whose average and peak values increased on Saturdays.	The first limitation concerns the wear time of accelerometers by PwMS. PwMS wore accelerometers on average one hour less than their healthy peers during weekdays. Second, the seven-day evaluation was a good compromise between the representativeness and feasibility of accelerometer measures. Third, it is essential to note that the analysis was done from a physical point of view and does not consider the physiological demands of groups. Finally, these results correspond to those obtained in a relatively small French population.
Salvi et al. [20]	The results demonstrated that app-based outdoor 6MWTs in community settings are valid, repeatable, and well accepted by patients. Its use should be made explicit to patients to increase their engagement, and it could complement or potentially substitute conventional 6MWTs in clinics.	This pilot study involved 30 patients and was not aimed at obtaining statistical significance. However, it is also important to observe that the manual data quality assurance strategy was inconsistent. As a result, an underestimated number of outdoor tests performed in the wrong conditions were included in the statistics.
Tan et al. [21]	The results indicated that five consecutive days of acute intermittent hypoxia (AIH) + WALK improved walking performance in persons with chronic, incomplete spinal cord injuries (SCI). The most significant change in performance occurred in persons who walk overground using bilateral arm-driven walking aids.	Many study limitations were due to the inherent heterogeneity of spinal cord injury in humans and the small sample size that precluded the generalization of the current findings. In addition, it focused on intralimb assessments of only the more impaired lower-limb and did not assess the less impaired lower-limb or interlimb coordination. Confounding factors likely contribute to the variability in responsiveness to AIH. Factors such as pre-existing medical conditions and delivery methods may influence the dose and timing of AIH.
Carommi et al. [22]	Results demonstrated that maximum gait speed is slightly lower in non-disabled people with multiple sclerosis (PWMS) than controls and that this finding is associated with poorer stability of gait, as indicated by a more significant local dynamic stability (LDS, e.g., short-term Lyapunov’s exponents, sLyEs) of gait. Furthermore, the correlation between vertical, mediolateral, and anteroposterior acceleration (sLyEAP) and the clinical measures of gait, balance, and fatigue suggests that sLyEAP could represent a measure of the stability of gait in PWMS valid from a clinical point of view.	sLyEVT and sLyEML (local dynamic stability was quantified by computing the short-term Lyapunov exponent (sLyE) from the trunk (i.e., lower back) anteroposterior (AP), mediolateral (ML), and vertical (VT) accelerations) are different in patients and controls. Still, neither is associated with any clinical measure of balance, gait, or impact of the disease. Moreover, lower trunk acceleration along the mediolateral axis is often proposed as a marker of gait impairment, and it might thus surprise that sLyEML does not correlate with clinical measures. However, sLyEs of acceleration provides different information (focused on local dynamic stability and amplitude) than the acceleration itself.
Gulart et al. [23]	Results demonstrated that the cut-off point of 28% was sensitive and specific to discriminate the functional status of patients with chronic obstructive pulmonary disease (COPD). Therefore, the %total score of the London Chest Activity of Daily Living (LCADL) reflects better outcomes of chronic obstructive pulmonary disease when compared to the total score.	One limitation is the lack of significance in the receiver operating characteristic curve analysis using the 6MWT as the anchor. In addition, some analyses have presented low statistical power, especially for the subgroups, since a sample estimation was not conducted for this purpose.
Paradiso et al. [24]	Results demonstrated that the Mi Band 2 is a suitable tool to measure steps at a moderate pace in healthy adults. However, the Mi Band device underestimates steps at lower speeds and heart rate during exercise.	First, the sample size of this study is restricted to healthy adults; thus, this limited the generalizability of their findings. Second, it assessed the Mi Band device under rest and exercise conditions in controlled laboratory settings. Still, it did not measure the device’s accuracy and reliability in free-living conditions. Last, the measured reliability between devices worn on the left and right wrists, but it did not measure reliability over time.
Plotnik et al. [25]	Results show that gait is more asymmetric and less coordinated as the disease progresses. Participants with mild multiple sclerosis (MS) showed significantly better bilateral coordination of gait (BCG) as reflected by lower e phase coordination index (PCI) values in comparison to the other two MS severity groups. BCG shows weaker clinical MS status associations than those observed between functional and subjective gait assessments and MS status. Like different neurological cohorts, BCG could be necessary to assess and target interventions among pwMS.	While providing data from a relatively large cohort of pwMS, the small number of participants in the severe group should be acknowledged as it limits the external validity of the findings. Additionally, gait asymmetry (GA) and BCG are worse in pwMS who have more disability and disease progression than those with mild to moderate disability.
Schubert et al. [26]	Results indicate that solely determining overall physical activity is not sufficient to predict exercise capacity. Only with quantification of the specific time spent in moderate activity, six-minute walk test outcomes were effectively predicted.	In this study, to determine resting heart rate, activity sensors data of the device were used. The paroxysmal occurrence of tachycardic atrial fibrillation, widespread in the older population, could potentially influence the heart rate-based analysis of activity. In addition, the influence of age, medication, and physical fitness on the resting heart rate could be a limitation for categorizing activity based on heart rate data.
Yeo et al. [27]	Results obtained indicate changes in diffusion tensor imaging (DTI) parameters in the vestibular neural pathway and are associated with age-related reductions in balance ability. The study provides primary data that could be used to plan exercise programs designed to mitigate fall risk.	The first limitation of this study is that the small number of subjects recruited limits the generalizability of its findings. Second, it was challenging to locate regions of interest precisely because of the diminutive sizes of vestibular nuclei. Third, it was not considered neuroplasticity because the subjects of this study were healthy adults.
Zeitlberger et al. [28]	Results of three cases of this study demonstrated that the 6WT is a helpful tool to assess outcome and monitor a patient’s functional walking capacity before and after lumbar epidural steroid injection (ESI). It further demonstrates that self-measurements of the 6WT are feasible in clinical practice and are well-accepted by patients.	The main limitation of this study is that the case series had a small sample size, which prevents meaningful statistical analysis. Therefore, further investigations to assess the validity and sensitivity of the 6MWT and the 6MWT’s ability to detect changes in the functional capacity of patients undergoing spine surgery or ESI are warranted inadequately powered studies.
Beausoleil et al. [29]	Results indicated that minimum toe clearance (MinTC) and stance phase variability along the 6MWT were significantly different. In addition, cadence variability and speed variation were significantly different between both feet (amputated and non-amputated leg). The increased variability in gait parameters along the 6MWT suggests a greater risk of future mobility problems following a return in the community. The data provided by the inertial sensors (IMUs) reflect the potential of the clinical rehabilitation program and could, therefore, help clinicians refine their interventions.	The small number of participants and the heterogeneity of the sample relating to age, sex, level, cause of amputation, and the number of days spent in a rehabilitation program limit the possibility of generalizing those outcomes.
Bertuletti et al. [30]	Results showed good accuracy in detecting steps with half the errors in detecting the step of the instrumented side compared to the non-instrumented. This test is the suggested configuration for patients walking with a large base of support	N/D
Camp et al. [31]	This study showed moderate to strong validity with measures of physical function, specifically 6MWD and gait velocity. In this study of chronic obstructive pulmonary disease patients, the Morton Mobility Index (DEMMI) was found to have good convergent, discriminant, and known-group validity related to measures of observed physical function and is an appropriate measure of mobility for physiotherapists’ and other healthcare professionals use in the acute care setting.	This study was conducted with younger patients (mean age 60 years) than typical AECOPD populations. Although the mean DEMMI score was like other reports, this younger group may be more mobile than older patients, and a sampling bias may be present. In addition, some patients who met the initial criteria of the study were discharged before day 3, also indicating a less severe AECOPD cohort. The sample size was calculated based on the validity analysis, which required a sample of 19 individuals for adequate power. However, the possibility of a DEMMI ceiling effect in this group could not be confirmed or denied in this study.
Moumdjian et al. [32]	Results demonstrated that all participants synchronized to both stimuli, yet multiple sclerosis (PwMS) synchronized better to music. Overall, participants had lower cadence, speed, and stride length overall conditions, except for HC, with increasing cadence during the music condition. In addition, PwMS perceived less cognitive fatigue, no difference in perceived physical fatigue, and a higher motivation walking to music than metronomes and silence.	One of the methodological limitations was that the walking track was 4.5 by 6 m square, and it is considered a limitation to this study as it induced many turns.
Taborri et al. [33]	This paper proposed synthetic indices to objectively measure the effects of prolonged walking on gait kinematics when multiple sclerosis patients performed prolonged walking. Proposed indices reveal themselves as reliable and repeatable both intra-day and inter-day. This fundamental metrological aspect allows assessing the evolution of a range of motion, gait variability, and gait asymmetry during a 6MWT as useful indices for monitoring gait deterioration.	N/D
Tousignant et al. [34]	Results demonstrated that most participants tended to improve their physical capacities such as walking distance and lower limb muscular strength. In addition, the use of sensors allowed a safe environment for the patient and an adequate and personalized exercise prescription.	The small number of participants is a weakness and a reality in pilot studies: no generalization is allowed.
Acuña et al. [35]	Results demonstrated considerable heterogeneity in performance on clinical balance and gait assessments. Abnormal muscle activation patterns were significantly correlated with variations in the dynamic gait index among the traumatic brain injury (TBI) subjects. Individuals who have experienced a prior TBI exhibit characteristic changes in the temporal coordination of select lower extremity muscles, which may contribute to impairments during challenging walking tasks.	The first limitation of this study is that the healthy controls were not matched for age, height, or weight. It could not be excluded that these differences may account for some of the observed differences between the TBI and healthy control subjects. Second, here was used the ensemble average muscle activation obtained over multiple strides, rather than individual stride data. Lastly, this study analyzed the activation of each muscle independently.
Byrnes et al. [36]	Results showed that the attractor for acceleration gait data varies largely among healthy subjects, and hence a reference attractor cannot be defined. Moreover, the change in the attractor and its variability during the 6MWT differed between patients and elderly healthy persons but not between repeated assessments. Attractor based on low-pass filtered signals could reflect pathology-specific differences in gait characteristics but does not appear to be sufficiently sensitive to serve as outcome parameter of decompression surgery in patients with symptomatic lumbar spinal stenosis.	A lack of statistically significant differences in exercise-induced changes in acceleration patterns and variability over time may be attributed to the heterogeneity of our patient population.
D’Alessando et al. [37]	Results demonstrated that older seniors showed lower serum albumin, hand-grip strength, body mass index (BMI), skeletal muscle mass, and resting energy expenditure. Protein intake was significantly lower in older seniors, whereas energy intake was similar. Average daily physical activity was lower in the older seniors than in the younger ones. Sarcopenia was more prevalent in older than in younger seniors. Among older seniors, sarcopenic and non-sarcopenic ones differed in age and performance on the six-minute walk test. In contrast, the estimated glomerular filtration rate (eGFR), biochemistry, dietary protein, and energy intakes were similar.	This study has several limitations: it is based upon a convenience sample of relatively limited size and involves only males. Another limit is choosing a creatinine-based formula (CKD-EPI) for estimated glomerular filtration rate calculation. The use of cystatin C-based eGFR might be preferable in this setting, avoiding an overestimation of eGFR in sarcopenic subjects.
Hadouiri et al. [38]	Results showed that after vascularized fibula free flap (VFFF) harvesting, patients have an altered walking capacity during prolonged walking conditions, which could be associated in part with the VFFF harvesting. The VFFF group in this study walked a reduced distance in the 6MWT, had an increased toe in/out angle on the operated side, and demonstrated altered physical exercise management during this lengthy walk task.	The main limitation is that the functional alterations observed in the VFFF group may have resulted from the VFFF. In addition, eight of the 11 patients in this study came from a typical population of smokers (including four with a drinker profile) who presented with maxillofacial or oral cancer. Another limitation concerns the small size of the study population.
El Hosainy et al. [39]	This study found a marked increase in serum lactate dehydrogenase levels associated with a significant decrease in serum creatine phosphokinase levels in idiopathic pulmonary fibrosis (IPF) patients compared to the control subjects that may reveal the presence of adaptive mechanisms in those patients to prevent muscle pain and fatigue. Additionally, a marked decrease in the mean of the serum IGF-1 levels in the newly diagnosed IPF patients compared to the control group, while the mean of the serum IGF-1 Levels in the previously diagnosed IPF patients was non-significant when compared to the other two groups. It may suggest a functional improvement with the start of steroid treatment.	N/D
Kennedy et al. [5]	Results indicated that children with Charcot–Marie–Tooth (CMT) were less active than typically developing (TD) controls. The children with CMT had a moderate disability and reduced ambulatory capacity in a six-minute walk test. Physical activity correlated with more significant disability and normalized six-minute walk distance-related disability affects physical activity and gait-related function in children and adolescents with CMT compared to TD peers. In addition, reduced physical activity adversely affects function across the timespan of childhood and adolescence into adulthood in people with CMT.	The limitations of this study included the relatively small participant numbers, broad age range, and mix of CMT subtypes which may have increased the variability of the results. Given the small sample numbers, it could not demonstrate differences in gait speed between CMT subtypes. Further, while lower limb surgery and injury over the 12-month interval were accounted for, physical activity levels and the occurrence of physiotherapy were not documented and may have influenced change over time. Low physical activity and exercise levels may contribute to gait and physical function deterioration and should be addressed in future studies.
Moon et al. [40]	Results demonstrated that BioStampRC sensors accurately and precisely measure gait parameters in multiple sclerosis (PwMS) across diverse walking impairment levels and detected differences in gait characteristics by disability level in PwMS. This technology can provide granular monitoring of gait both inside and outside the clinic.	A primary limitation is that 6MW trials were conducted on a motorized treadmill. It is established that treadmill walking is distinct from overground walking. The treadmill was utilized to maintain gait speed within a given trial. Additionally, while the inertial sensors provide tri-axial angular velocity and acceleration data, the study only utilized angular velocity data to analyze temporal gait parameters. Further, the different devices used were not attached to the body’s exact location. While this helped minimize interference between devices, there might be some errors due to the other attachment locations.
Toosizadeh et al. [41]	Results show promise of a quick upper-extremity measure of functional capacity in patients with chronic obstructive pulmonary disease (COPD, and as an outcome measure in clinical COPD trials. Speed, power, and moment upper-extremity function parameters were independently associated with 6MWD when controlling for age, gender, and body mass index. In addition, elbow moment showed significant Pearson correlations with all pulmonary function measures and maximal inspiratory/expiratory pressure measures.	As with measurement limitations for gait-based measures, upper-extremity disability or injury may limit upper-extremity function (UEF) measurement. Further, due to the small sample size, current results should be considered preliminary and need further validation among larger samples. Additionally, a small percentage of participants were at the severe COPD stage, and therefore, the current sample may not adequately represent those with functional/gait impairments. Also, the study lacks intra- and inter-rater reliability assessments. Lastly, although a strong correlation was observed between UEF and 6MWD tests, the association between UEF outcomes and long-term prospective clinical measures, including the risk of exacerbations, hospitalization, and mortality, should be assessed.
Cheng et al. [42]	Results showed that every patient now has correct modeling of GOLD level, even sample by sample (10 s), not only walk test by walk test (6 min). Measuring motion is a potential solution for passive monitoring, distinguishing this work from the phone applications measuring pulmonary functions with the microphone. A passive monitor has compliance advantages over active phone applications. The patient simply uses their phone as usual during daily activities. No particular actions or special experts are necessary.	N/D
Cheng et al. [43]	Results demonstrated that a universal model, trained with about sixty patients 6MWT data, has perfect accuracy at detecting all statuses from GOLD 0 to GOLD 3. In addition, it gives a way to interpret better how the predictive model works and how the input features, containing demographics and gait features, interact with each other.	N/D
Kalron [44]	Results demonstrated that the walk ratio discriminates between multiple sclerosis (MS) fallers and non-fallers and between different neurological disability levels. In addition, the consistency of the walk ratio results between the instrumental and manual measurements is encouraging due to the measurement’s clinical feasibility and applicability that can be provided either with or without instrumental gait measurement devices.	Walk ratio was not sensitive to asymmetries between left and proper steps. The impact of gait asymmetry on the walk ratio measure is unclear in the neurological population. The study did not provide data on the walk ratio application in MS using a walking assistive device. All outcome scores were based on a single measurement session, preventing from concluding the reproducibility of the walk ratio measure over time.
Qureshi et al. [12]	Results illustrated that the variables from a severely impacted gait stand out as a subject completes the test. Minute-based extension of the feature space is promising for personalized signal processing by letting physicians observe changes in gait parameters over time instead of actual values to remove inter-patient variability.	N/D

## Data Availability

Not applicable.
